# Targeting the Substrate in Ablation of Persistent Atrial Fibrillation: Recent Lessons and Future Directions

**DOI:** 10.3389/fphys.2018.01158

**Published:** 2018-09-18

**Authors:** Martin K. Stiles, Prashanthan Sanders, Dennis H. Lau

**Affiliations:** ^1^Waikato Clinical School, University of Auckland, Hamilton, New Zealand; ^2^Department of Cardiology, Waikato District Health Board, Hamilton, New Zealand; ^3^Centre for Heart Rhythm Disorders (CHRD), South Australian Health and Medical Research Institute (SAHMRI), The University of Adelaide and Royal Adelaide Hospital, Adelaide, SA, Australia

**Keywords:** atrial fibrillation, ablation techniques, lifestyle interventions, mapping & localization, fibrosis, imaging, three-dimensional, imaging

## Abstract

While isolation of the pulmonary veins is firmly established as effective treatment for the majority of paroxysmal atrial fibrillation (AF) patients, there is recognition that patients with persistent AF have substrate for perpetuation of arrhythmia existing outside of the pulmonary veins. Various computational approaches have been used to identify targets for effective ablation of persistent AF. This paper aims to discuss the clinical aspects of computational approaches that aim to identify critical sites for treatment. Various analyses of electrogram characteristics have been performed with this aim. Leading techniques for electrogram analysis are Complex Fractionated Atrial Electrograms (CFAE) and Dominant Frequency (DF). These techniques have been the subject of clinical trials of which the results are discussed. Evaluation of the activation patterns of atria in AF has been another avenue of research. Focal Impulse and Rotor Modulation (FIRM) mapping and forms of Body Surface Mapping aim to characterize multiple atrial wavelets, macro-reentry and focal sources which have been proposed as basic mechanisms perpetuating AF. Both invasive and non-invasive activation mapping techniques are reviewed. The presence of atrial fibrosis causes non-uniform anisotropic impulse propagation. Therefore, identification of fibrosis by imaging techniques is an avenue of potential research. The leading contender for imaging-based techniques is Cardiac Magnetic Resonance (CMR). As this technology advances, improvements in resolution and scar identification have positioned CMR as the mode of choice for analysis of atrial structure. AF has been demonstrated to be associated with obesity, inactivity and diseases of modern life. An opportunity exists for detailed computational analysis of the impact of risk factor modification on atrial substrate. This ranges from microstructural investigation through to examination at a population level via registries and public health interventions. Computational analysis of atrial substrate has moved from basic science toward clinical application. Future directions and potential limitations of such analyses are examined in this review.

## Introduction

While isolation of the pulmonary veins (PVI) is firmly established as effective treatment for the majority of paroxysmal atrial fibrillation (AF) patients,(Calkins et al., 2017) there is recognition that patients with persistent AF have substrate for perpetuation of arrhythmia existing outside of the pulmonary veins. Computational approaches have been attempted in the search to identify targets for effective ablation of persistent AF. We aim to discuss the clinical aspects of computational approaches that seek to identify critical sites for ablation in the treatment of persistent AF. We explore the initial approach of electrogram-based analyses through to more topical panoramic mapping of AF substrate. We look at the role of imaging to identify atrial scar as a potential AF ablation target and the recent recognition that lifestyle management is very important in reducing AF burden. Lastly, we explore potential future directions to advance AF care via computational approaches.

### Electrogram-based approach

#### Complex fractionated atrial electrograms

Electrophysiologists skilled in ablation of arrhythmias have sought to look for characteristics of electrograms that might identify critical sites for ablation. The elimination of complex fractionated atrial electrograms (CFAE) has been shown in some studies to be an effective strategy of catheter ablation (Nademanee et al., 2004, 2008) Fractionated or prolonged electrograms have been demonstrated to identify areas acting as pivot points, slowed conduction, anisotropy, localized circuits or rotors, all of which are capable of sustaining re-entry (Spach and Dolber, 1986; Konings et al., 1994; Haïssaguerre et al., 2006). Accurately identifying such electrograms may allow targeted ablation to halt wavelet re-entry and prevent the perpetuation of AF.

Initially, CFAE were defined as fractionated electrograms composed of ≥2 deflections, perturbation of the baseline with continuous deflection of a prolonged activation complex, or atrial electrograms with a cycle length ≤ 120 ms (Nademanee et al., 2004) However, as this method of ablation became more widespread, a more consistent definition of ablation targets was desired, particularly if one wanted to perform a multi-center trial where standardization across hospitals was paramount. Hence, computer algorithms were designed to provide consistent definitions of CFAE, independent of the operator's discretion. These included the CFAE software module (CARTO, Biosense Webster, CA, USA) and the CFE-mean tool (NavX, Abbott, CA, USA).

The CFE-mean tool was used to test the usefulness of CFAE ablation in addition to PVI and linear lesions in the BOCA study of 130 persistent AF patients (Wong K. C. et al., 2015) This trial found no additional benefit in the patients randomized to CFAE ablation, despite longer procedure and ablation times. There was an excess of organized atrial arrhythmia, in particular gap-related macro-re-entrant flutter, in the patients randomized to CFAE ablation. In the multi-center STAR-AF II trial (Verma et al., 2015) the use of a computerized algorithm to detect standardized CFAE electrograms was employed. This trial showed no additional benefit of CFAE ablation as guided by the CFE-mean tool in addition to PVI alone, in patients with persistent AF. Indeed, some have used this trial to suggest that CFAE-targeted ablation is detrimental to long-term outcome (Conti and Verma, 2016) The CHASE-AF trial produced similar conclusions, where no benefit from the addition of a CFAE-based ablation strategy over non-CFAE ablation was seen (Vogler et al., 2015) A recent meta-analysis confirmed that the addition of extra-pulmonary substrate ablation such as that of CFAE in persistent AF patients, was associated with declining efficacy as compared to PVI ablation alone (Clarnette et al., 2017).

However, strong proponents of CFAE-based ablation argue that computer-derived 3D maps of fractionation are inaccurate and lead to different areas being targeted, compared to the areas targeted when visual recognition of CFAEs are made by experienced operators (Oketani et al., 2016) For example, the semi-automated CFAE algorithms from the NavX and CARTO systems were found to correlate poorly with each other or AF complexity measures that may explain the variable results of CFAE-based ablation. Furthermore, the dynamic nature of CFAE with poor temporal stability may be another challenge for CFAE-based ablation based on point-by-point mapping (Lau et al., 2012) Novel indices such as unipolar fractionation index or spatiotemporal stability index of CFAE have been shown to demonstrate superior physiological relevance to AF dynamics (Lau et al., 2015; Thanigaimani et al., 2017a) Therefore, there perhaps remains a role for novel computerized algorithms used in combination with higher density mapping catheter that could better direct electrophysiologists to critical AF-sustaining sites.

#### Dominant frequency

Dominant frequency (DF) analysis aims to distill the local activation frequency from highly complex electrograms. This utilizes computer algorithms (usually fast fourier transform) to assign a fundamental frequency of electrical activation. The DF can then be displayed on a 3D map to guide the ablator to sites of high DF thought to be driving the AF (focal source or rotor). The aim of such analysis is to detect sites of high frequency that have been hypothesized to “drive” the fibrillation process (Jalife et al., 2002) These sites have been shown by retrospective analysis to identify effective ablation areas (Sanders et al., 2005) In an elegant animal study by Kalifa et al, areas of fractionation were demonstrated at the periphery of areas of high dominant frequency (Kalifa et al., 2006) The proximity of high DF and CFAE sites has also been demonstrated in high-density mapping of human AF (Stiles et al., 2008) Of note, most studies examining DF guided ablation have used off-line analysis, although real-time analysis has also been reported albeit without incremental outcome (Atienza et al., 2014) In a systematic review of DF-based approaches, Gadenz et al concluded that DF-based approaches are a useful marker of ablation outcome; however, direct intervention targeting DF sites appears premature with mixed results and too few studies (Gadenz et al., 2017) A more recent study using a novel frequency analysis algorithm and longer duration of AF electrograms in search for temporally stable AF drivers has shown some promise (Kimata et al., 2018) Ongoing work will help refine our armamentarium toward future targeting of high DF sites to improve outcomes (Sanders et al., 2018).

#### Shannon entropy

The detection of points of high Shannon Entropy has been postulated as a way of mapping drivers of AF (Ganesan et al., 2014) In particular, ablation at the point of “phase singularity” has been shown to lead to termination of atrial fibrillation (Narayan et al., 2012) Recordings at the center of a rotor should have less directional information in the local bipolar electrogram than recordings away from the center. Maximum Shannon Entropy has been shown to be co-located at the center of rotational activity from experimental models of atrial fibrillation (Ganesan et al., 2014) Furthermore, Shannon Entropy of bipolar electrograms has been shown to be consistent across models and differences in electrode spacing, signal filtering and rotor meander (Ganesan et al., 2014) Studies examining ablation outcome based on Shannon Entropy guided ablation are ongoing.

With the number of promising computational approaches seeking to gather additional information from electrograms, some have sought to show which technique is best, or whether a hybrid approach combining multiple approaches is superior. Hwang et al looked at phase singularity, DF, Shannon entropy and CFAE cycle length with subsequent ablation in 2D and 3D simulation models and found that DF-based ablation was superior for AF termination (Hwang et al., 2016) However, no human AF studies have been able to replicate such data and it remains an area where computational based approaches to electrogram analysis may yet yield insights into effective ablation targets for human AF.

### Panoramic mapping of AF mechanisms

Over the last decade, the field has progressed from electrogram-based AF mapping to focus on activation and phase mapping to detect AF drivers in the form of rotational (“rotors”) and ectopic focal (“foci”) activations. First descriptions of rotational activations were from studies that undertook sequential mapping with multi-polar spiral catheter (Atienza et al., 2011; Ghoraani et al., 2013) The Focal Impulse and Rotor Modulation (FIRM) guided technique was the first panoramic mapping study that showed high success rates with ablating AF drivers (Narayan et al., 2012). Other panoramic mapping techniques included body surface potentials mapping with inverse-solution electrocardiographic imaging (ECGI) (Haissaguerre et al., 2014) mapping of wavefront propagation using intracardiac multipolar catheter (CARTOFINDER) (Honarbakhsh et al., 2018) and non-contact mapping using a multielectrode array catheter (ENSITE) (Yamabe et al., 2016; Lee et al., 2017). Table 1 summarizes the different panoramic mapping techniques utilized toward detection of AF rotors and foci.

**Table 1:** Clinical panoramic mapping for AF rotors and foci.

	**FIRM**	**ECGI**	**CARTOFINDER**	**Ensite**
Mapping tool	64-electrode Basket type catheter	252-electrode body surface vest	20-electrode PentaRay or 64-electrode Basket type catheter	Ensite multielectrode array catheter
Contact mapping, Surface	Yes, Endocardial	No, Epicardial	Yes, Endocardial	No, Endocardial
Mapping window, Electrogram	Unknown, Unipolar and Bipolar	9 s, Unipolar surface potentials	30 s, Unipolar	7.5 s, Virtual unipolar
Signal processing algorithm	Phase mapping (proprietary)	Wavelet and phase mapping (proprietary)	Phase or Activation mapping	Activation mapping only
No. of persistent AF patients studied (n)	>500	>100	33	30
Key Findings	AF rotors/foci are sustained Variable outcomes from meta-analysis	AF Rotors/foci are non-sustained Outcome data from single-centre only	Rotors/foci are non-sustained Variable processing algorithms and limited outcome data	Limited evidence of transient rotors No outcome data to date

#### Focal impulse and rotor modulation (FIRM)

The FIRM technique is facilitated by a 64-pole basket type catheter with phase-based signal processing to detect AF rotors or foci. The algorithm has remained proprietary, with utilization of electrogram data such as local refractoriness and restitution. The initial study reported a mean of 2.1 sources (rotors or foci) per patient that conserved for tens of minutes. In this study, FIRM-guided ablation was superior to conventional ablation with 86 vs. 20% acute termination or slowing of AF, and 82.4% vs. 44.9% freedom from AF after a median of 9 months (Narayan et al., 2012). Further, follow-up of these patients to 3 years showed durable success with 77.8% of the FIRM-guided ablation group remained free of AF vs. 38.5% in the conventional group after a mean of 1.2 procedures (Narayan et al., 2014).

However, subsequent studies have shown a wide variability in the reported outcomes from FIRM-guided ablation. A recent meta-analysis (10 studies, *n* = 527 patients) showed a pooled estimate of single-procedure freedom from AF of only 59.2% in non-paroxysmal AF at a mean follow-up of 12.9 months, with a high level of heterogeneity seen among studies (*I*^2^ = 88.3%) (Parameswaran et al., 2018). The variability in outcomes could be due to the limitations in the use of the basket type catheter including suboptimal electrode contact or chamber coverage where potential absence of septal coverage and <25% of overall left atrial surface area coverage have been reported (Pathik et al., 2018). The assumption that the electrodes of the basket catheter are evenly spread over a 2-D grid as opposed to the actual variable spread in a 3-D orientation may contribute to potential errors in phase-based signal analysis of focal sources (Pathik et al., 2018). Further, it is unclear whether electrode density or spacing of the basket catheter may also affect detection of focal sources (Walters et al., 2016; Kuklik et al., 2017). There are also concerns regarding the validity of the FIRM technique whereby comparative assessments failed to identify the same temporally stable rotors as identified by FIRM, with absence of distinctive electrophysiological characteristics of rotors in terms of dominant frequency and Shannon Entropy (Benharash et al., 2015; Halbfass et al., 2017). Several ongoing randomized trials will provide further guidance on the utility of FIRM guided ablation.

#### Electrocardiographic imaging (ECGI)

The ECGI is a non-invasive body surface potentials mapping technique using a 252-electrode vest with inverse solution to derive virtual potentials on the epicardial atrial surface localized with thoracic computed tomography. Additional signal processing of wavelet transform and phase mapping are then applied to detect AF sources. Initial report of ECGI mapping in 26 AF patients found mainly multiple wavelets and ectopic foci with rare rotor activities seen in 15% only (Cuculich et al., 2010). More recent ECGI mapping study of 103 persistent AF patients showed a median of 4 driver regions per patient that consisted of non-sustained repetitive rotors (median 2.6 rotations) with substantial meandering as well as ectopic focal sources that fired a mean of 6 times. In this study, the rotors accounted for 80.5% of all AF drivers with the remaining 19.5% consisted of ectopic foci. Importantly, ablation of these drivers resulted in AF termination in 80% and in these cohort of patients, AF freedom was 85% at 1 year follow-up (Haissaguerre et al., 2014). In addition, the ECGI data from the same group also demonstrated increased complexity of these AF drivers with prolonged AF duration. Specifically, longer duration of AF was associated with increased numbers of rotors and ectopic foci, increased number of regions with these AF drivers and extrapulmonary drivers, such as from the infero-posterior left atrium and the anterior left atrium/septal region. Ablation targeting these driver sites resulted in AF termination in 70% of the persistent AF patients (Lim et al., 2017). However, there are several shortcomings with this mapping modality such as the inability to detect activations in the interatrial septum or the ridge between the pulmonary vein and left atrial appendage, reduced ability to detect low amplitude signals as well as the inability to distinguish between micro-entry and epicardial breakthrough activations (Cuculich et al., 2010; Haissaguerre et al., 2014).

#### CARTOFINDER

This is a mapping approach that utilizes existing CARTO 3-D electroanatomical mapping system (Biosense Webster, CA, USA) with a module called CARTOFINDER which is still in the development phase. The first report using this system included 13 persistent AF patients where mapping was performed with the PentaRay catheter in both the right and left atrium. In brief, it utilizes unipolar electrograms for phase analysis using Hilbert Transform to detect rotors as well as bipolar electrograms for dominant frequency analysis to gauge ablation efficacy. A mean of 1.8 rotor domains (mean 9.2 rotations) was seen in each patient while ablation of these sites resulted in reduction in dominant frequency, acute termination to sinus rhythm in 2 out of 13 patients (15%) and 1-year freedom from AF rate of 70% (Calvo et al., 2017). In another CARTOFINDER study, activation mapping was performed with the basket catheter in 20 persistent patients without using phase based analysis. Here, the AF drivers were transient (mostly ≤ 4 cycles) but repetitive in separate maps while ablation of these resulted in significant effect of termination or slowing of cycle length in 85% (Honarbakhsh et al., 2018). More outcome data are awaited as this system matures in its development.

#### Ensite non-contact multi-electrode array

Several groups have used the Ensite multi-electrode array catheter (St Jude Medical, MN, USA) for panoramic non-contact AF mapping (Yamabe et al., 2016; Lee et al., 2017). This is a commercially available system that affords recording of unipolar virtual electrograms that can be superimposed onto the 3-D endocardial geometry to display wavefront propagation as animated isopotential color map. Using this technique, transient AF rotors were seen in 1 out of 15 persistent AF and 10 out of 60 paroxysmal AF patients that lasted for a mean of 6.1 s in one study (Yamabe et al., 2016). In another study, the Ensite non-contact mapping system failed to identify any focal sources in 15 persistent AF patients (Lee et al., 2017). There are no data available regarding ablation of AF rotors or foci detected with this system.

#### Electrophysiological characterization of AF drivers

The various panoramic mapping methods described above represent intensive research in the field in search of AF driver sites. Many studies have provided increasing insights regarding the electrophysiological characteristics of these drivers despite apparent differences seen in the dynamics of AF drivers. For example, all panoramic mapping modalities have found AF driver sites to be non-sustained except for the FIRM technique where AF drivers lasted for tens of minutes. The transient nature of AF drivers is in keeping with direct contact mapping studies in long-lasting persistent AF patients (Lee et al., 2014; Walters et al., 2015). Nevertheless, there appears to be agreement on the meandering nature of these drivers that appear repetitively at similar locations in the same patient, namely near the pulmonary vein ostia, left atrial appendages, septum and coronary sinus-inferior left atrium. The anatomical clustering of AF drivers is in keeping with the known importance of structures annexed to the left atrium and the presence of complex muscle fiber orientations at such sites (Haïssaguerre et al., 2005).

Further analysis of the ECGI detected driver sites have unveiled increased electrogram fractionation and their proximity to areas with increased fibrosis as assessed by late gadolinium-enhanced magnetic resonance imaging (LGE-MRI) (Haissaguerre et al., 2016). However, others have not been able to show the same relationship between AF drivers and LGE-MRI detected fibrotic regions (Chrispin et al., 2016; Sohns et al., 2017). Nevertheless, novel 3-D computational framework provides evidence that AF drivers may be identifiable by a distinct structural “fingerprints” that consist of intermediate wall thickness, intermediate fibrosis and twisted myofiber orientation (Zhao et al., 2017). Taken together, further work is needed to refine our understanding of AF drivers and resolve the differences seen in the their dynamics to guide ablative therapy. The non-disclosures of proprietary algorithms in the detection of AF drivers may be a major obstacle toward rapid translation into clinical practice. Advancement in catheter technology and design to afford better chamber coverage with higher electrode density will also aid in the search of AF drivers amidst the irregularly irregular atrial activations of this highly complex arrhythmia.

### Targeting atrial fibrosis

Atrial fibrosis is known to result in non-uniform anisotropic impulse propagation and increased conduction heterogeneity that may perpetuate AF by favoring re-entry and anchoring of AF drivers (Maesen et al., 2013; Haissaguerre et al., 2016). These structural and conduction changes have been consistently seen in different atrial substrates such as hypertension, obesity, heart failure, valvular heart disease, diabetes, aging and obstructive sleep apnea (Sanders et al., 2003; Kistler et al., 2004; Kato et al., 2006; John et al., 2008; Lau et al., 2010, 2011a, 2013b; Medi et al., 2011; Dimitri et al., 2012; Abed et al., 2013a; Iwasaki et al., 2014). In addition, AF itself can result in increased atrial fibrosis in the absence of any risk factors (Stiles et al., 2009; Verheule et al., 2013; Corradi et al., 2014). The signaling mechanisms involved in atrial fibrosis are highly complex and remain incompletely understood (Thanigaimani et al., 2017b). Several agents have been studied in experimental models and have been found to be effective in attenuating or preventing atrial fibrosis: renin-angiotensin-aldosterone inhibitors,(Li et al., 2001; Milliez et al., 2005) n-3 polyunsaturated fatty acids, (Lau et al., 2011b) HMG-CoA reductase inhibitors,(Shiroshita-Takeshita et al., 2007) and various anti-fibrotics such as tranilast, pirfenidone and relaxin (Lee et al., 2006; Nakatani et al., 2013; Parikh et al., 2013; Henry et al., 2016). Unfortunately, human studies of these agents remain lacking for translation into clinical practice.

**Figure 1:**
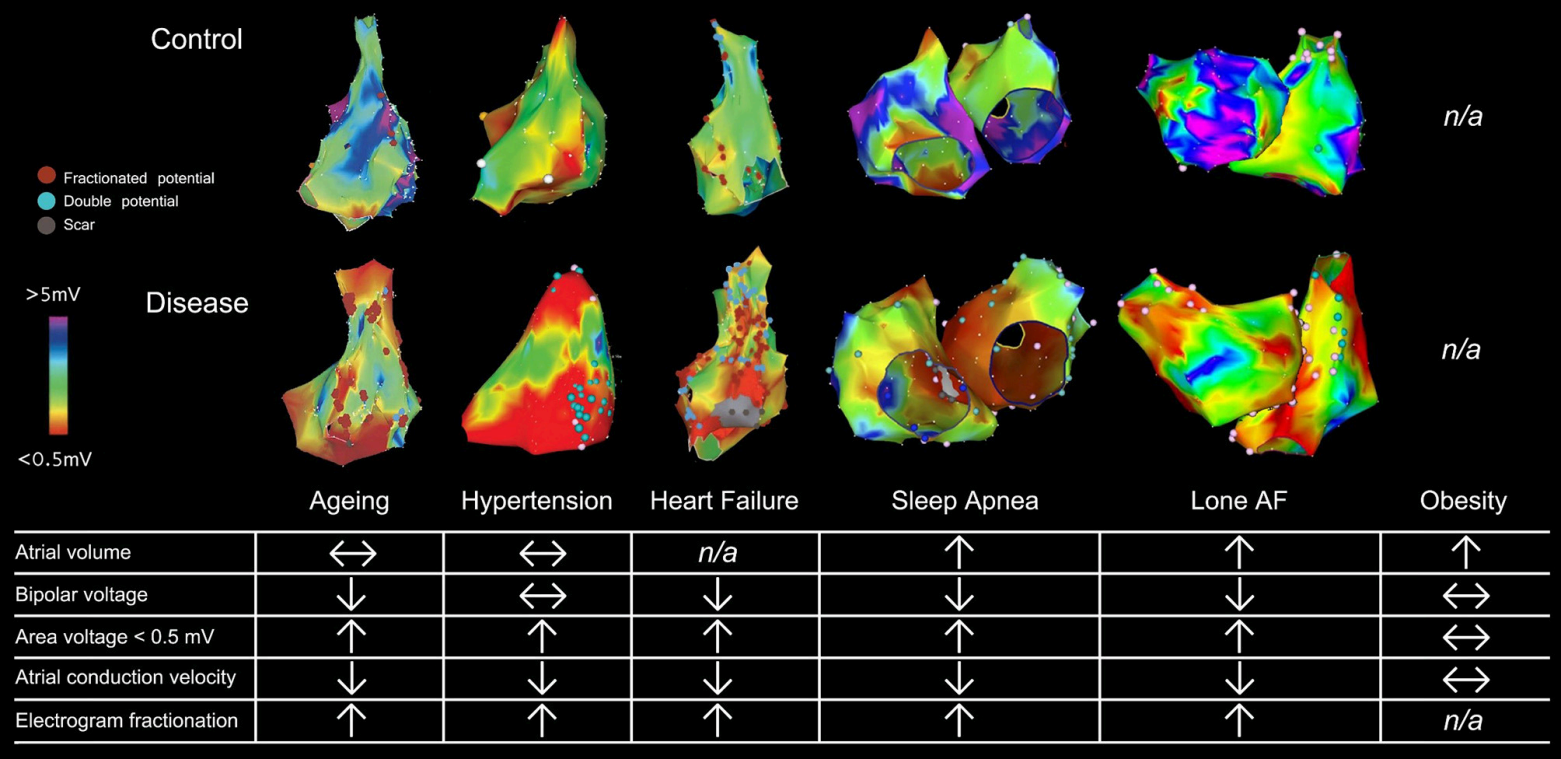
Electroanatomical maps and electrophysiological changes in various AF substrates. 3-D electroanatomical maps from various AF substrates are shown with bipolar voltage scaled from <0.05 mV (red) to >5 mV (purple). Points with fractionated or double potentials or scar are annotated with red, blue, and gray dots respectively. Figure used by permission from Lau et al. (2017) © American Heart Association.

Traditional assessment of atrial electrical changes has been facilitated by 3-D electroanatomical maps to evaluate atrial voltage, conduction velocity and electrogram fractionation (Figure 1; Lau et al., 2017). Advances in cardiac imaging have facilitated non-invasive quantification of atrial fibrosis by means of LGE-MRI (Oakes et al., 2009). Atrial fibrosis assessed with LGE-MRI has been shown to associate well with regions of low bipolar left atrial voltage as determined by 3-D electroanatomical systems (Malcolme-Lawes et al., 2013; Zghaib et al., 2018). In addition, it has been demonstrated that the degree of atrial fibrosis detected by LGE-MRI increased with AF persistence and the presence of more AF risk factors (Daccarett et al., 2011; McGann et al., 2014). Importantly, atrial fibrosis defined by LGE-MRI has been shown to be independently associated with AF recurrence in patients undergoing catheter ablation in the delayed-enhancement MRI determinant of successful radiofrequency catheter ablation of AF (DECAAF) study (Marrouche et al., 2014). Further analysis of 177 of the DECAAF patients who underwent repeat LGE-MRI scanning 90 days post-ablation showed that the greater overlap of ablation induced scarring over pre-ablation fibrosis, the better the arrhythmia free survival (Akoum et al., 2015). Ongoing prospective multi-center randomized controlled trial (DECAAF-II) will examine the efficacy of targeting LGE-MRI detected atrial fibrosis in persistent AF patients.

However, several technical challenges with LGE-MRI detection of atrial fibrosis have been acknowledged. These include issues such as spatial resolution given the thin atrial walls, motion artifact especially when the patient is not in sinus rhythm, the lack of standardized image acquisition protocols and the all-important quantitation of the amount of LGE that can be subjective and labor intensive even if signal intensity thresholding was employed over visual assessment (Appelbaum and Manning, 2014; Pontecorboli et al., 2017). It is also noted that there remains a paucity of good quality data whereby LGE-MRI detected atrial fibrosis or post-ablation scars have been histologically validated (Harrison et al., 2014; McGann et al., 2014). Therefore, it is of no surprise that the reproducibility of LGE-MRI analysis has been questioned and the diagnostic accuracy of this modality to detect ablation lesions has been shown to be suboptimal (Hunter et al., 2013; Pontecorboli et al., 2017). Alternatively, post-contrast cardiac MRI atrial T1 relaxation time mapping has been shown to be a reliable index of atrial fibrosis that correlated with atrial voltage and ablation outcome (Ling et al., 2014). However, the inability of T1 mapping technique to provide spatial distribution of atrial fibrosis will hamper its ultimate usage to guide ablative therapy. Hopefully, the LGE-MRI technique will mature toward standardization of image acquisition, automation of image analysis, improved image resolution and validation in the not too distant future.

### Targeting the AF risk factors

Despite the advances in catheter ablation technology and strategies over the last two decades, the field has not witnessed a significant improvement in ablation success especially in those with persistent AF (Brooks et al., 2010; Clarnette et al., 2017). It is well recognized that there is a long-term attrition in sinus rhythm maintenance following initially successful catheter ablation (Ganesan et al., 2013). In addition, progressive atrial substrate changes have been documented in individuals despite a successful AF ablation procedure (Teh et al., 2012). A myriad of AF risk factors have been identified to contribute to the progressive AF substrate and recurrences post catheter ablation procedures. These include: aging, left atrial enlargement, heart failure, hypertension, aortic stiffness, valvular heart disease, obesity, pericardial fat, diabetes mellitus, dyslipidemia and obstructive sleep apnea (de Vos et al., 2010; Ng et al., 2011; Wong et al., 2011; Mohanty et al., 2012; Lau et al., 2013a; Jacobs et al., 2015; Proietti et al., 2015; Wong C. X. et al., 2015; Linz et al., 2018). Therefore, efforts must be placed to target these risk factors to maximize rhythm control outcome in patients with AF.

**Figure 2:**
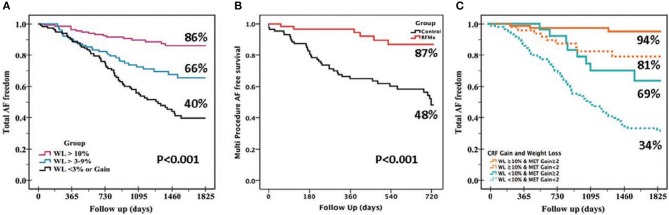
Beneficial effects of various lifestyle modifications. The benefits of lifestyle and risk factor modifications on AF-free survival are evident from these Kaplan-Meier survival graphs: **(A)** Greater freedom from AF was seen with greater degree of weight loss (WL) in the LEGACY study. **(B)** Risk factor management (RFM) confers greater AF-free survival following catheter ablation procedure vs. usual care in the ARREST-AF Cohort Study. **(C)** Gain in cardiorespiratory fitness (MET, metabolic equivalent) confers independent and incremental AF free survival to WL in the CARDIO-FIT study.

There is prospective randomized evidence showing that a targeted weight loss intervention reduced atrial dilatation, left ventricular hypertrophy, and AF symptom burden and severity in highly symptomatic overweight and obese patients with AF (Abed et al., 2013b). Tight control of systolic blood pressure to under 130 mmHg has been shown to reduce incident and recurrent AF in hypertensive subjects although a more recent study reported neutral results (Thomas et al., 2008; Okin et al., 2015; Parkash et al., 2017). Continuous positive airway pressure therapy has been shown to improve sinus rhythm maintenance in patients with obstructive sleep apnea undergoing electrical cardioversion and catheter ablation (Linz et al., 2018). A structured, physician-driven, and goal-directed weight and risk factor management strategy has been applied in overweight and obese patients with AF to good success, as seen in the ARREST-AF (Aggressive Risk Factor Reduction Study for Atrial Fibrillation and Implications for the Outcome of Ablation) and LEGACY (Long-Term Effect of Goal Directed Weight Management on an Atrial Fibrillation Cohort) studies (Figure 2). In brief, this program involved weight management by dietary modification, tailored moderate-intensity exercise to improve cardiorespiratory fitness, targeted strict systolic blood pressure control to <130 mm Hg, lipid and glycemic management aiming for low-density lipoprotein <2.6 mmol/L and hemoglobin A1c <6.5%, active screening and treatment of obstructive sleep apnea with continuous positive airways pressure therapy, smoking cessation and alcohol reduction to <3 standard drinks per week. In addition, gain in cardiorespiratory fitness was found to confer greater freedom from AF independent of weight loss.

Additional evidence on the benefits of exercise in AF can be seen from the study by Malmo and co-workers with aerobic interval training for 12 weeks (Malmo et al., 2016). Similarly, weight and exercise intervention has been shown to confer equivalent benefits to re-do catheter ablation in a study in patients with post-ablation AF recurrences (Mohanty et al., 2014). More recently, in persistent AF patients with heart failure, a multi-center prospective randomized study has demonstrated that additional intervention targeting the underlying conditions with cardiac rehabilitation including physical activity, dietary restrictions, and counseling, mineralocorticoid receptor antagonist, HMG-CoA reductase inhibitor and angiotensin converting enzyme inhibitors and/or receptor blockers resulted in improved sinus rhythm maintenance at 1 year (Rienstra et al., 2018). Taken together, the mounting evidence regarding the benefits of these lifestyle and risk factor management approaches and their cost-effectiveness warrants their incorporation as routine “4th pillar” of AF care to maximize outcomes (Lau et al., 2017; Pathak et al., 2017). This can be achieved by combining the risk factor management component with a wider integrated AF clinic to optimize care delivery (Hendriks et al., 2012).

### Future directions

Novel computational approaches that help identify the arrhythmogenic substrate of AF have the potential to advance the field of AF ablation. The progression of electrogram analysis through to dynamic substrate mapping has been increasingly reliant on the computational approach. Recognition that atrial microstructure is critical to the maintenance of persistent AF should stimulate future analyses to benefit from the increasing resolution of imaging studies, particularly MRI. However, with increasing resolution comes reliance on computing power and bespoke algorithms to take best advantage of it.

As we move into the world of meta-data and wearable technology, a computational approach to analysis of the data may afford increased detection of AF as well as giving additional insights to physical activity and its impact on AF incidence. Personalized exercise and weight reduction programmes for patients with AF delivered via smartphone technology will integrate with aggressive risk factor management clinics. Computational analysis of effectiveness and the consequent outcomes for AF will no doubt be an important future application.

## Conclusion

Recent developments in computational approaches to ablation of atrial fibrillation have focused on identifying drivers for the perpetuation of this arrhythmia. The future of detecting critical sites for ablation depends largely on a computational approach. However, large data analyses may also play a role in lifestyle adjustment which is now recognized to be an important part of a comprehensive patient management programme for AF.

## Author contributions

MS and DL wrote sections of the manuscript and proofread each other's section. PS proofread the manuscript and provided guidance on the overall direction of the manuscript. All authors critically appraised the final version of the paper.

### Conflict of interest statement

PS reports having served on the advisory board of Biosense-Webster, Medtronic, Abbott, Boston Scientific and CathRx. PS reports that the University of Adelaide receives on his behalf lecture and/or consulting fees from Biosense-Webster, Medtronic, Abbott, and Boston Scientific. PS reports that the University of Adelaide receives on his behalf research funding from Medtronic, Abbott, Boston Scientific, Biotronik and Liva Nova. DL reports that the University of Adelaide has received on his behalf lecture or consulting fees from St Jude Medical, Boehringer Ingelheim, Bayer, and Pfizer. The remaining author declares that the research was conducted in the absence of any commercial or financial relationships that could be construed as a potential conflict of interest.
